# Higher incidence of steroid-induced ocular hypertension in keratoconus

**DOI:** 10.1186/s40662-016-0035-9

**Published:** 2016-02-23

**Authors:** Anastasios John Kanellopoulos, Emerson M. Cruz, Robert Edward T. Ang, George Asimellis

**Affiliations:** Laservision.gr Clinical and Research Eye Institute, 17 Tsocha Street, Athens, Postal Code: 11521 Greece; NYU Medical School, New York, NY USA; Asian Eye Institute, Makati, Philippines

**Keywords:** Keratoconus, Intraocular pressure, Corticosteroid induced ocular hypertension, IOP spike, Corneal collagen cross-linking, CXL, The Athens protocol, Topography-guided, Photorefractive keratectomy, Dexamethasone topical ophthalmic solution

## Abstract

**Background:**

To compare intraocular pressure (IOP) changes following topical dexamethasone administration for 1 month in keratoconic versus normal eyes.

**Methods:**

This is a retrospective, single-center, non-randomized case series evaluation of 350 eyes. Two groups were formed: normal/control Group A (n_A_ =73), eyes that underwent excimer laser photorefractive keratectomy; and keratoconic (KCN) Group B (n_B_ =277), eyes that were subjected to partial laser photorefractive keratectomy combined with collagen cross-linking (The Athens Protocol). All eyes received the same post-operative regimen of topical dexamethasone 0.1 % for at least 1 month. Goldmann applanation tonometry IOP readings and central corneal thickness (CCT) measurements were monitored. Cases with induced ocular hypertension (OHT, defined as post-operative IOP higher than 21 mmHg), were identified and correlated to refractive procedure, gender, and corneal thickness.

**Results:**

At 4 weeks postoperatively, OHT was noted on 27.4 % (20 /73 eyes) in Group A, and 43.7 % (121 /277 eyes) in KCN Group B, (*p* <0.01). Six months post-operatively (following 5-months of discontinuing topical dexamethasone treatment and commencing treatment of IOP-lowering medications), OHT rate was 1.8 % in Group A and 3.9 % in the KCN Group B.

**Conclusion:**

This study demonstrates a potentially significant pre-disposition of keratoconic eyes to the development of steroid-induced OHT.

## Background

Keratoconus is usually a bilateral disease, characterized by progressive corneal thinning leading to ectasia [1]. It has been proposed that the decreased biomechanical strength in a keratoconic cornea may also be associated with reduced support for the optic nerve at the level of the lamina cribrosa, increasing glaucomatous damage susceptibility [2]. While accurate assessment of intraocular pressure (IOP) is imperative for early glaucoma detection, the asymmetrically astigmatic cornea in keratoconus [3] may pose challenges for proper IOP reading [4].

Collagen cross-linking (CXL) has been employed for stabilization of progressive keratoconus [5, 6]. Complications from this technique are rarely encountered [7–11]. CXL has been combined with customized anterior-surface normalization in a procedure introduced as the Athens Protocol (AP) [12]. The procedure involves combined, sequential excimer-laser epithelial debridement, partial, topography-guided photo refractive keratectomy (PRK), and high-irradiation CXL [13].

Topical corticosteroids are routinely prescribed after excimer-laser ablation procedures such as laser in situ keratomileusis (LASIK) and PRK due to their anti-inflammatory action. Use of topical corticosteroids is the most widely-studied risk factor for ocular hypertension (OHT), which is associated to increased IOP readings in some individuals known as steroid responders [14] or in glaucoma patients. It has been suggested that corticosteroid use activates molecular myocillin gene production [15], which alters trabecular meshwork morphology, severing aqueous flow facility [16].

On the other hand, it has been postulated [17] that increased IOP readings following CXL may be attributed to increased corneal rigidity [18]. It is unclear therefore, if such increased IOP readings correlate to increased ocular susceptibility to a corticosteroid regimen.

This study aims to investigate the 1-year postoperative changes in IOP in a large group of keratoconic patients subjected to the combined PRK and CXL procedure (AP) and to compare these IOP changes to those observed in non-keratoconic eyes subjected to the PRK procedure. To the best of our knowledge, this is the first study that investigates the incidence of OHT after partial PRK combined with CXL among keratoconic patients.

## Methods

This is a retrospective, single-center, non-randomized case series evaluation. We retrospectively identified the latest 350 cases that received either PRK or AP procedures at Laservision.gr Institute, and had a minimum of 3 months follow-up. The study was performed in accordance with the policies of the LaserVision.gr Institute Ethics Committee, which provided approval, and adhered to the tenets of the Declaration of Helsinki.

### Inclusion – exclusion criteria

The study evaluated the following two groups: Group A, serving as the control group included non-keratoconic eyes that underwent routine PRK for correction of myopic refractive error (73 eyes). The eyes included in this group had no pre-operative clinical or topographic evidence of keratoconus and are thus referred to as ‘normal’ eyes. Group B, serving as the study group included clinically established progressive keratoconus, managed with the AP (277 eyes). In cases of bilateral surgery, only one eye was included per patient (randomly selected).

Inclusion criteria for the study for either groups were successful and uncomplicated intervention (PRK in Group A and AP in Group B). Exclusion criteria for the study for either groups were preoperative IOP >21 mmHg, family history of glaucoma, corneal opacity, previous ocular surgery, present or history of herpetic keratitis, active ocular infection, autoimmune disease, chemical injury, history of delayed epithelial healing, and age less than 18 years. Neither group involved pregnant or lactating female patients at the time of the intervention. No glaucoma diagnosis or predisposition to glaucoma (pigment dispersion, pseudoexfoliation, abnormal gonioscopy, irregularity of the optic nerve) was observed pre-operatively. A complete and comprehensive ophthalmic evaluation was performed in all patients in all groups prior to the surgical intervention. This evaluation included the possibility of family history of glaucoma, complete biomicroscopical evaluation to include gonioscopy, IOP measurement by Goldmann applanation tonometry, and careful assessment of the optic nerve during dilated-pupil fundus evaluation. As with all evaluations in our center, they were recorded digitally to become available for future review.

### Surgical procedures

The PRK procedure involved epithelial removal over an 8-mm central corneal area, facilitated by 20 % ethanol solution applied for 30 s. The myopic excimer-laser stromal ablation was then performed. A corneal shield soaked with mitomycin-C 0.02 % was placed over the ablation zone for 20 s and then irrigated with chilled balanced salt solution (BSS).

The AP procedure also involved surface ablation. The epithelium was first removed via excimer-laser debridement. The subsequent excimer-laser stromal ablation was based on topographic cornea data supplied by Scheimpflug tomography or Placido topography [19]. Mitomycin-C (0.02 %) was applied for 20 s followed by copious irrigation with 50 cc of cold (4 °C) BSS. Dextran-free 0.1 % riboflavin solution was instilled for 5 min over the ablated cornea. High-irradiance ultraviolet-A (10 mW/cm^2^) collagen cross-linking was applied for 10 min.

Following either procedure (PRK or AP), a bandage soft contact lens was placed and was removed typically on the fourth post-operative day. All procedures were performed by the same surgeon (AJK).

All eyes in both groups received the same post-operative medication: topical moxifloxacin hydrochloride ophthalmic solution 0.5 % (Vigamox, Alcon, Fort Worth, TX) four times a day for 1 week; fixed combination of dexamethasone 0.1 % with chloramphenicol (Dispersadron C, Novartis, Basel, Switzerland) three times a day for 1 month, then tapered to twice a day until consume. In addition, autologous serum was prescribed three to four times a day until consume to treat dry-eye related problems.

In cases identified at the one month post-operative visit with IOP reading more than 21 mmHg, an IOP-lowering treatment plan was implemented depending on the magnitude of IOP increase. The initially-prescribed topical corticosteroids were discontinued and topical glaucoma agents were administered: timolol maleate 0.5 % ophthalmic solution (Timoptic, Merck, Whitehouse Station, NJ), topical dorzolamide 2 % solution (Trusopt, Merck, Whitehouse Station, NJ), or their combination (preservative-free Cosopt). These cases were closely monitored and attended an unscheduled eye examination within 7 to 14 days. Topical anti-glaucoma regimen was maintained until the IOP returned to near (less than 3 mmHg difference) pre-operative levels.

### Data collection

Patient data were queried for the following information: age, gender, date of treatment and eye treated; IOP (reported in mmHg); and central corneal thickness (CCT) (reported in μm). IOP and CCT were recorded pre-operatively, 1-month, 3-months, 6-months and 12-months post-operatively. CCT was measured via Scheimpflug topometry (Pentacam, Oculus Optikgeräte GmbH, Wetzlar, Germany).

The IOP was measured by the same investigator (AJK) via Goldmann applanation tonometry (GAP) during the slit-lamp examination. We did not adjust IOP readings for corneal thickness, potentially erring towards underestimating actual IOP. For the purpose of this study, we considered cases with ocular hypertension (OHT, or IOP spike) using the widely-accepted definition of an IOP reading >21 mmHg [20]. As ‘responders’, we considered the cases with increased post-operative IOP, in comparison to pre-operative.

Statistical analysis was performed by Graph Pad Statistical Calculator (GraphPad Software Inc, San Diego, CA). Values are reported as mean ± standard deviation (range, minimum to maximum). The Fisher exact and chi-square tests were used; *p* value equal to or less than 0.05 was considered to assess statistically significant differences. Univariate regression statistical analysis was performed to determine the association of clinical variables with postoperative OHT.

## Results

Table 1 shows patient demographics and pre-operative clinical data for each group. Pre-operative mean IOP in Group A was 13.417 ± 3.01 mmHg (10 to 21 mmHg) and in KCN Group B 13.422 ± 2.38 mmHg (8 to 20 mmHg), *p* =0.792. Both groups had long-term follow-up. Average follow-up time was 7.28 ± 4.83 months in Group A and 8.12 ± 5.19 months in Group B. At 3-months, all cases were fully evaluated (73 eyes in Group A and 277 eyes in Group B). Of those cases, complete data were available from 20 eyes in Group A and 121 eyes in Group B, the reason being that additional follow-up was required due to a possible increase in IOP noted early postoperative interval (1-month).

**Table 1:** Data per treatment group: number of eyes, age, gender, pre-operative and 1-month postoperative central corneal thickness (CCT) and intraocular pressure (IOP)

Parameters	Group A (Control)	Group B (KCN)	*p* value
Number of operated eyes (n)	73	277	
Age (Years)	36.59 ± 13.50	30.01 ± 12.58	0.064
	range 18 to 77	range 18 to 58	
Gender (Female: Male)	25 : 14	73 : 119	
Pre-operative CCT(μm)	508.70 ± 44.46	456.62 ± 56.82	<0.01
	range 371 to 611	range 287 to 549	
One-month postoperative CCT (μm)	421.91 ± 51.09	365.95 ± 64.24	<0.01
	range 319 to 582	range 202 to 527	
Pre-operative IOP(mmHg)	13.417 ± 3.01	13.422 ± 2.38	0.792
	range 10 to 21	range 8 to 20	
One month post-operative IOP (mmHg)	17.42 ± 3.60	22.15 ± 6.42	<0.001
	range 11 to 24	range 12 to 51	

*CCT=* central corneal thickness, *IOP=* intraocular pressure, *KCN*= keratoconic

**Fig. 1:**
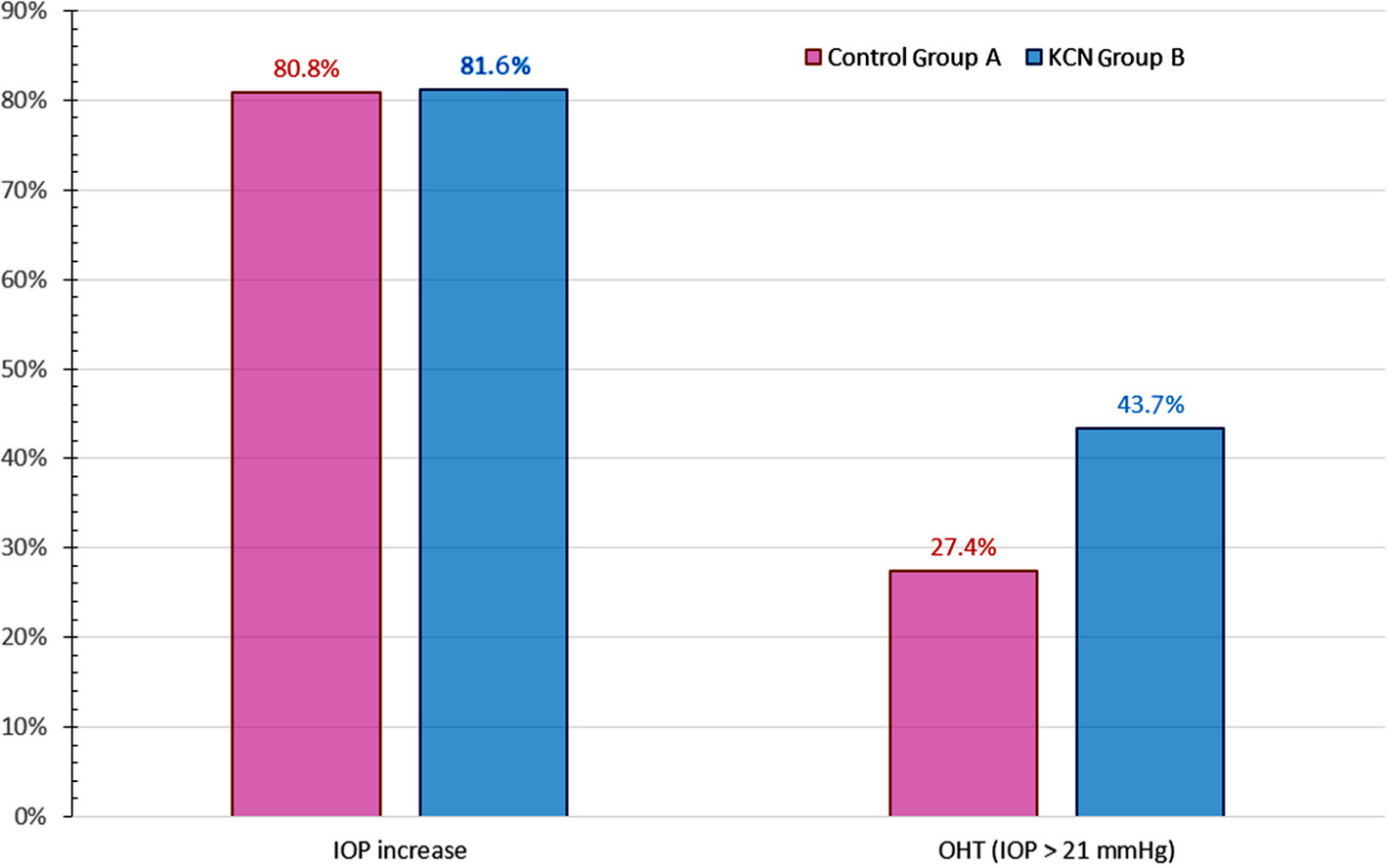
Rate of steroid-induced intraocular pressure (IOP) increase and ocular hypertension (OHT) rate observed 1 month post-operatively. OHT is defined as IOP >21 mmHg

One month post-operatively, a statistically significant difference in the incidence of OHT was noted between the two groups (*p* <0.01), (Table 2 and Fig. 1). In Group A, OHT occurred in a total of 20 out of 73 eyes (27.4 %) vs. the keratoconic Group B in a total of 121 out of 277 eyes (43.7 %).

**Table 2:** Incidence of intraocular pressure (IOP) postoperative increase and ocular hypertension (OHT, defined as IOP >21 mmHg) by treatment group

	IOP increase incidence	OHT incidence
Control Group A	59/73 eyes (80.8 %)	20/73 (27.4 %)
KCN Group B	226/277 eyes (81.6 %)	121/277 (43.7 %)
*p* value	0.951	<0.01

*IOP=* intraocular pressure, *OHT=* ocular hypertension, *KCN=* keratoconic

**Fig. 2:**
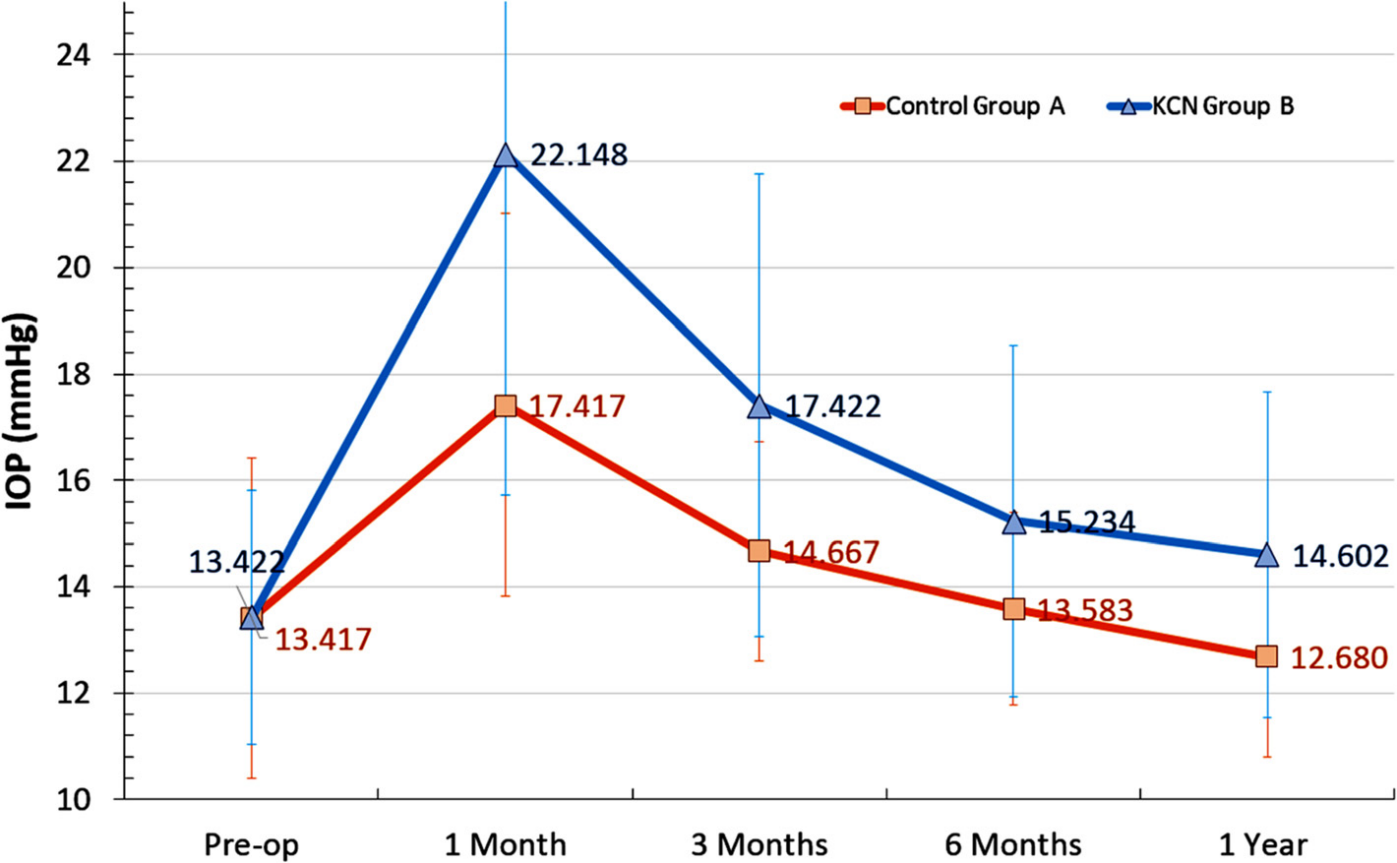
Longitudinal development of IOP between control Group A and keratoconic Group B

In Group A, the 1-month post-operative IOP increase from pre-operative was +4.00 ± 4.15 mmHg (−4 to +12 mmHg), while in Group B it was +8.726 ± 6.70 mmHg (−4 to +38 mmHg (*p* <0.001)). A longitudinal plot of the IOP values (up to 12 months) is illustrated in Fig. 2. One year post-operatively, mean IOP in Group A was 12.68 ± 1.89 mmHg and in Group B 14.60 ± 3.09 mmHg. The difference between 1-year post-operative to pre-operative IOP for Group A was −0.737 mmHg (*p* =0.039), and for the keratoconic Group B, +1.18 mmHg (*p* =0.017). Between the two groups, there is a statistically significant difference in IOP when comparing any period, except for the pre-operative baseline (*p* =0.792, <0.001, 0.003, 0.007 and 0.012, respectively for pre-operative and 1-month, 3-months, 6-months, and 12-months post-operative periods).

Once OHT was noted, low-dosed corticosteroid drops alone were prescribed in 11/20 eyes (55 %) in Group A, compared to 62/121 eyes (51.2 %) in Group B. Low-dosed corticosteroid drops plus anti-glaucoma treatment were prescribed in Group A in 25 % (5/20 eyes) and in Group B in 32 % (39/121 eyes). Anti-glaucoma treatment alone was prescribed in Group A in 20 % (4/20 eyes) and in Group B in 16.5 % (20/121 eyes).

In nearly all cases, IOP returned to lower readings within 1 week, except for a small subset in the keratoconic Group B. At 3 months post-operatively, 3 eyes (2.7 %) in Group B were continually observed and prescribed anti-glaucoma treatment until IOP returned to pre-operative levels (with a margin of +3 mmHg). No eyes in Group A were further observed at this time point. Until completion of all visits, IOP in both groups was within the pre-operative levels. No case in any group progressed to advanced glaucoma.

**Fig. 3:**
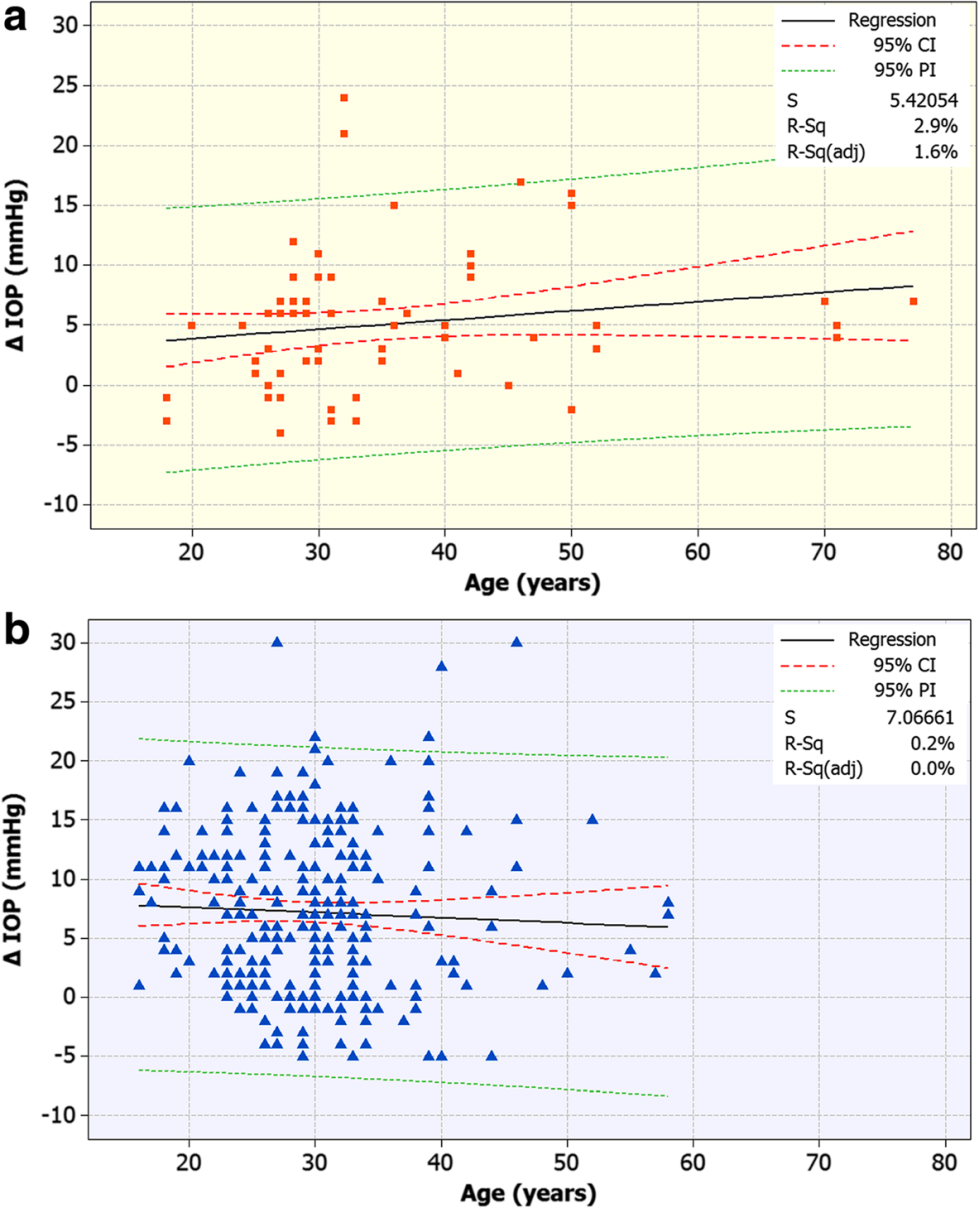
Linear regression of intraocular pressure (change of IOP reading with respect to age). *Top*, control Group A, *bottom*, KCN Group B. (r^2^ = 0.029, *p* <0.10, Group A, and r^2^ = 0.002; *p* < 0.82, Group B)

Univariate linear regression analysis of pre-operative age and CCT with respect to 1-month detected IOP change was performed. We investigated the following correlations between magnitude of IOP increase (difference 1-month post-operatively to pre-operatively) and age (Fig. 3), gender, pre- and post-operative CCT, and difference (reduction) in CCT. Age and gender did not indicate any correlation to IOP increase in either group. Strong casual correlation reached statistical significance only for postoperative CCT in relation to IOP increase (r^2^ = 0.12; *p* <0.01 Group A; r^2^ = 0.078, *p* <0.02 KCN Group B). Within KCN Group B, results showed that male gender poses a higher risk than female (*p* <0.001); age did not appear to be a factor; and pre-operative CCT less than 450 μm has a higher risk for developing OHT (odds ratio 0.578, *p* =0.89), while post-operative CCT did not show to be a distinguishing factor. Table 3 summarizes the chi square results and concomitant *p* value for each pairwise comparison.

**Table 3:** Association of ocular hypertension (OHT) occurrence with Age, Gender, and pre-operative central corneal thickness (CCT). Odds ratio calculated as conditional maximum-likelihood estimate (cMLE). Confidence limits are reported as the Mid-P exact

Parameters	Odds ratio	95 % Confidence interval	*P* value
Gender Male	1.45	0.46–1.45	0.58
Gender Female	2.50	0.94–6.69	0.10
Age below 50 years	1.77	0.80–3.91	0.18
Age above 50 years	1.25	0.06–26.87	0.99
CCT ≤450 μm	0.5787	8.58-undefined	0.89
CCT >450 μm	0.1875	0.052–0.541	0.00

*CCT=* central corneal thickness

## Discussion

The findings observed in our study merit attention in the sense that keratoconic eyes displayed a different behavior in IOP increase following a refractive procedure such as AP (a combination of partial PRK with accelerated CXL), as compared to normal, non-KCN eyes that underwent a PRK refractive procedure. These two groups of patients were compared because of the similar refractive ablation procedure and the similar post-operative corticosteroid regimen prescribed. Among the demographic variables recorded, an age difference was noted (*p* =0.064) with Group A consisting of older patients than the KCN Group B. This is justified by the fact that progressive keratoconus is manifested in young adults, whereas myopia correction is an option for candidates over a wide range of age. In addition, in the KCN Group B, there was a notable higher incidence of males, consistent with our clinical experience in male/female ratio in keratoconic patients [21], and keratoconus incidence studies [22]. Our analysis however, showed that gender did not impose a greater OHT independent risk factor.

The keratoconic eyes evaluated in this study showed higher IOP values throughout the 12 month follow-up period in comparison to the non-KCN eyes. These differences were statistically significant even at the 12 month post-operative visit, but were more evident (*p* <0.001) at the first post-operative month visit (Fig. 2). At 1 month post-operatively, all patients in both groups were receiving corticosteroids treatment, whereas no patient from either group was on such regimen at the 12 month visit. It is notable that while in both groups the pre-operative IOP did not have any statistically significant difference, 1-year postoperatively, the difference of +1.18 mmHg of the KCN group was statistically significant (*p* =0.017), in agreement with other studies [23].

There are three main factors that should be considered in order to explain the findings in different IOP changes observed in these two groups:

The first factor is cornea thinning resulting from either procedure. Several studies have investigated the effect of CCT on IOP readings and there is consensus in the literature that thinner corneas result in lower IOP readings [24, 25]. If the theory of IOP reduction were to be attributed to this corneal thickness reduction, one would expect lower IOP measurements at the 12-month visit in both, not just the ‘control’ group. However KCN eyes showed higher IOP values throughout the post-operative period compared to pre-operative, while the ‘control’ eyes had initial increase, which tapered to a statistically significant lower average IOP 12-months post-operatively. Thus, we postulate that corneal thickness reduction has a minimal (if any) impact on the comparative results we observed in this study. Corneal thickness reduction alone may not explain the difference between IOP changes between the two groups.

A second factor influencing IOP measurements is corneal rigidity. The improved biomechanical properties (corneal strengthening) associated with the CXL part of the AP procedure (Group B) affect the applied force necessary to flatten the cornea [26] and are predisposed to higher IOP readings (overestimation). If the differences in IOP measurements between the two groups had been the same throughout the 1-year follow-up period, we could have attributed these differences solely to corneal rigidity. However, the IOP findings have extreme statistical significance at 1 and 12 months between the two groups. The 12-month IOP difference between the two groups (+1.92 mmHg, *p* =0.017) could to some extend be attributed to the CXL effect on IOP; however, at the first post-operative month, the IOP difference between the two groups was much larger (+4.73 mmHg, *p* <0.001). Therefore, other factors must be considered to explain the longitudinal IOP differences observed, particularly those observed at 1-month.

The use of topical corticosteroids during the first post-operative month is a factor, therefore, that may explain the findings. Topical corticosteroid use has routinely been employed after refractive procedures in order to suppress inflammation. One disadvantage of topical corticosteroid use is the induction of OHT. It was observed in the early 1960s that 30 % of clinically ‘normal’ human eyes could have steroidal response upon 6 weeks exposure to topical application of corticosteroids [27]. OHT has been observed in the immediate post-operative period as a result of corticosteroid regimen following corneal keratoplasty [28–31], LASIK [32], and PRK [33]. Of the two procedures involving excimer laser ablation, LASIK [32] and PRK are associated with transient post-operative OHT with incidence estimates from 8 to 32 % [33]. Several mechanisms have been suggested as causative factors such as inflammation and potential steroid response. Accumulation of extracellular material in the juxtacanalicular connective tissue region and between extracellular trabecular beams in these steroid- treated eyes [34] as well as thickening of the trabecular beams and an activation of trabecular cells have been reported. Moreover, it has been suggested that the glucocorticoid-induced deposition of myocillin in the extracellular material of the trabecular meshwork leads to increased aqueous humor outflow resistance [16]. However, the total clearance time of the glucocorticoid induced myocillin deposition is undocumented.

Data in our study indicate that after 1 month of topical corticosteroid treatment, 43.7 % of KCN patients appeared to be steroid responders compared to 27.4 % of the non-KCN, ‘normal’ patients. Availability of data regarding OHT incidence among keratoconic patients is scarce. Table 4 summarizes the incidence of OHT in related published studies involving normal patients subjected to refractive procedures. Based on our findings, we could either postulate that KCN eyes exhibit a higher ‘concentration’ of myocillin, and/or a higher incidence of myocillin polymorphisms [35]. Although the latter needs to be confirmed through genetic studies [36], the myocillin deposition theory might also be reinforced by the riboflavin/UV combination effect. Diffusion of riboflavin into the anterior chamber results in possible outflow of riboflavin molecules via the trabecular meshwork. Cross-linking at this point may partially contribute to alteration of the trabecular meshwork structure and affect its biomechanical properties, negatively affecting outflow facility. The aforementioned theories remain to be proved as they entail a long-term and meticulous follow-up.

**Table 4:** Intraocular pressure (IOP) response to topical corticosteroid administration found in literature and our current study

Study	Type of Subjects	Number of subjects	Corticosteroid	Incidence (≥21 mmHg)
Armaly [34]	Normal	-----	Dexamethasone	36 %
Becker [27]	Normal	50	Betamethasone	30 %
Jain [38]	Normal	50	Betamethasone	41 %
Javadi [33]	PRK	327	Betamethasone	7.9 %
Gatry [39]	PRK	120	Dexamethasone	12 %
Seiler [40]	PRK	146	Dexamethasone	26–32 %
Frucht-Pery [41]	LASIK	1,492	Prednisolone acetate	0.06 %
Our Current Study	Normal	73	Dexamethasone	34.24 %
Our Current Study	Keratoconus	277	Dexamethasone	48.37 %

*PRK=* photo refractive keratectomy, *LASIK=* laser in situ keratomileusis

In summary, this study identified a significant higher proportion of keratoconic eyes (43.7 %, *p* <0.001) that developed OHT, in relation to non-keratoconic eyes. The observed IOP differences were significant in the first post-operative month, during which all eyes were treated with topical corticosteroids. This may suggest that keratoconic eyes when treated with CXL behave as steroid responders more often than non-keratoconic eyes. Possible mechanisms for that have been discussed. The findings in the study should caution clinicians in close monitoring of IOP changes related to potential topical (ocular, nasal, inhalers) [37] or systemic corticosteroid use, that are commonly administered in the traditionally allergy-prone and/or atopic keratoconus population.

## Conclusions

Decreased corneal thickness (such as encountered in keratoconus and post refractive surgery patients) is associated with lower IOP readings. This poses a major concern for early glaucoma detection because the true IOP is grossly underestimated. This study indicates that keratoconus may be a risk factor for steroid induced ocular hypertension. All clinicians administering topical corticosteroids for eye, nasal, bronchial and pulmonary use should be aware of such a potential.
